# Dynamics of Immune Checkpoints, Immune System, and BCG in the Treatment of Superficial Bladder Cancer

**DOI:** 10.1155/2017/3573082

**Published:** 2017-10-26

**Authors:** Farouk Tijjani Saad, Evren Hincal, Bilgen Kaymakamzade

**Affiliations:** Department of Mathematics, Near East University, North Nicosia, Northern Cyprus, Mersin 10, Turkey

## Abstract

This paper aims to study the dynamics of immune suppressors/checkpoints, immune system, and BCG in the treatment of superficial bladder cancer. Programmed cell death protein-1 (PD-1), cytotoxic T-lymphocyte-associated antigen 4 (CTLA4), and transforming growth factor-beta (TGF-*β*) are some of the examples of immune suppressors/checkpoints. They are responsible for deactivating the immune system and enhancing immunological tolerance. Moreover, they categorically downregulate and suppress the immune system by preventing and blocking the activation of T-cells, which in turn decreases autoimmunity and enhances self-tolerance. In cancer immunotherapy, the immune checkpoints/suppressors prevent and block the immune cells from attacking, spreading, and killing the cancer cells, which leads to cancer growth and development. We formulate a mathematical model that studies three possible dynamics of the treatment and establish the effects of the immune checkpoints on the immune system and the treatment at large. Although the effect cannot be seen explicitly in the analysis of the model, we show it by numerical simulations.

## 1. Introduction

Cancer is a class of diseases characterized by out-of-control cell growth which affects and damages the DNA. Cancer prevalence is increasing in many countries [1]. Many treatment options of cancer exist, which include surgery, immunotherapy, chemotherapy, radiotherapy, vaccine therapy, and hormonal therapy [1, 2]. The mode and type of treatment depend on the type, location, and grade of the cancer and the patient's body. The bladder is a hollow organ in the lower abdomen which collects urine produced by the kidneys. Bladder cancer is a growth of malignant cells initiating in the urinary bladder. It is common, with around 38,000 men and 15,000 women diagnosed every year in the United States. Approximately 400,000 new cases are diagnosed and about 150,000 die directly from the disease every year across the globe [3, 4].

The bladder wall is lined with cells called transitional and squamous cells. The most common type of bladder cancer is urothelial carcinoma or transitional cell carcinoma (TCC). It mostly originates from the transitional cells and further progresses and grows superficially on the inner surface of the bladder; as a result, it invades the bladder wall and vessels, dispersing into the neighboring organs as well as forming distant metastases [5–7].

One of the most effective ways of treating bladder cancer is immunotherapy. This is the process of stimulating, activating, and triggering the immune system to spread, locate, and kill cancer cells [8].

Intravesical Bacillus Calmette-Guerin (BCG) is an attenuated nonpathogenic strain of* Mycobacterium bovis* that was initially used as a vaccine against tuberculosis. The attenuation was reached via manipulation of the bacillus by serial growth on a culture medium. As a result, the genes causing virulence will be lost and inoculated into humans [9, 10]. It is undoubtedly the most efficient and successful immunotherapy of cancer [9]. BCG therapy is used for various types of cancers, including acute lymphoblastic leukemia and melanoma. The first report of successful use of BCG to treat patients with bladder cancer was in 1976 by Morales et al. They obtained the efficacy of BCG therapy and established it as the pillar for the treatment of non-muscle-invasive bladder cancer after transurethral resection [5, 11].

Intravesical BCG is a type of immunotherapy that is also used to treat superficial bladder cancer [12, 13]. It is usually applied after local surgery to prevent tumor recurrence. It is given in 6-weekly intravesical instillation of 1.5 × 10^8^ bacteria, which has been proven to be superior to chemotherapy in reducing recurrence rates of the tumor [12–14]. When the BCG is instilled and processed into the bladder, it creates an inflammatory environment which in turn stimulates an immune response, resulting in attacking the cancer cells. Therefore, many researchers believed that BCG reduces tumor progression and stated that the primary role of BCG treatment is to stimulate, trigger, and activate the immune effector cells in order to attack the cancer cells. In spite of the fact that BCG instillation is regarded as the “gold standard” treatment, it has many side effects which include hematuria, pain, dysuria, and fever, to mention a few [7–14].

Immune checkpoints are negative regulators of the immune system which play important roles in maintaining self-tolerance, preventing autoimmunity, and protecting tissues from immune collateral damage. These immune checkpoints are often hijacked by tumors to restrain the ability of the immune system to mount an effective antitumor response. The tumors neutralize some immune checkpoint pathways in order to maintain immune resistance, particularly against T-cells. The T-cells are specific tumor antigens. Examples of the aforementioned checkpoints are PD-1 and CTLA4 [15–17].

Programmed cell death protein-1 (PD-1) is a protein that is encoded by the PDCD1 gene in humans. It is a cell surface receptor which belongs to the immunoglobulin superfamily and is expressed on T-cells and pro-B-cells. PD-1 binds two ligands, PD-L1 and PD-L2. The PD-1 acts as an immune checkpoint, which plays an important role in downregulating the immune system by preventing the activation of the T-cells. Hence, it decreases autoimmunity and encourages self-tolerance [18, 19]. The immune system is directly affected by the activities of PD-1 in the sense that it suppresses, blocks, and deactivates the immune cells from spreading, fighting, and attacking the cancer cells. Therefore, PD-1 aids in growth, development, and progression of the cancer. In conclusion, it disrupts and affects immunotherapy [20–24].

Transforming growth factor-beta 1 (TGF-*β*1) is a regulatory cytokine which suppresses immune function in cancers and in chronic viral infections. It inhibits the activation of the T-cells and subdues their proliferation. Hence, cancer cells take advantage of this immune checkpoint pathway as a way to escape and evade detection. This leads to the inhibition of antitumor immune response, resulting in cancer growth and development [25, 26].

Mathematical modeling and simulation helps in predicting treatments' outcome, as well as describing the behavior and complex dynamics involved. Bunimovich-Mendrazitsky et al. (2007, 2008, and 2011) modeled mathematically the use of BCG in noninvasive bladder cancer, where their study identified fixed points and conditions for stability of the dynamical system [6, 8, 14]. In 2016, Bunimovich-Mendrazitsky developed a new mathematical model for combined BCG and IL-2 bladder cancer treatment which introduces the effect of TAA T-cells. Furthermore, Starkov utilized a mathematical approach for bladder cancer treatment model in the derivation of ultimate upper and lower bounds. He also presented tumor clearance conditions for BCG treatment of bladder cancer [13].

In this research, we formulate a mathematical model to study the dynamics of immune checkpoints/suppressors, immune system, and the BCG immunotherapy of bladder cancer. Moreover, we highlight the effects of immune checkpoints/suppressors on the immune system and the treatment numerically.

This paper is organized as follows.  Section 1 is the introduction.  Section 2 is the formulation and presentation of our model. We give the stability analysis and numerical simulations in Sections 3 and 4, respectively. In the final section, we state our conclusions and discussions.

## 2. Formulation of the Model

The model consists of a system of four nonlinear differential equations, which characterize the dynamics of the interaction between cancer cells (*C*), different arms of the immune system regarded as effector cells (*E*), the BCG (*B*), and all categories of immune suppressors/checkpoints as (*P*).

### 2.1. Dynamics of Cancer Cells

The dynamics of cancer cells is given by

(1) $$\begin{array}{c} \frac{dC}{dt}=rC-\frac{\alpha_{1}EC}{P+k}. \end{array}$$

Here, we assume that, in the absence of the immune system, the cancer cells grow exponentially with growth rate *r*. The second term shows the elimination of cancer cells by the effector cells at the rate *α*_1_, while 1/(*P* + *k*) is the immunosuppressive factor by the immune checkpoints/suppressors, which interrupts the activities of the effector cells, with *k* being an inhibitory parameter.

### 2.2. Dynamics of the Effector Cells

The dynamics of the effector cells is given by

(2) $$\begin{array}{c} \frac{dE}{dt}=\frac{a_{1}CE}{P+k}+\frac{a_{2}BE}{P+k}-\alpha_{2}EC-\mu_{1}E. \end{array}$$

The first term here gives the recruitment of effector cells at the rate *a*_1_ which is directly proportional to the population of cancer cells (i.e., occurring due to the direct presence of cancer cells). *a*_2_*BE* shows the activation of effector cells by BCG at the rate *a*_2_. *a*_1_ is the antigenicity of cancer cells which triggers an immune response in the host. It is believed that the immune checkpoints will distort both the recruitment and the activation of effector cells; hence, 1/(*P* + *k*) is the immunosuppressive response which puts a limitation on the recruitment level and interrupts the activation of effector cells, with *k* here being an inhibitory parameter. The next term gives the elimination of effector cells by the cancer cells at the rate *α*_2_, and the last term describes the degradation of effector cells at the rate *μ*_1_.

### 2.3. Dynamics of BCG

The dynamics of BCG is given by

(3) $$\begin{array}{c} \frac{dB}{dt}=b-\alpha_{3}EB-\mu_{2}B. \end{array}$$

The first term *b* is the constant rate of introduction of BCG into the bladder, the second term describes the elimination of BCG by effector cells at the rate *α*_3_, and the third term gives the decay of BCG at the rate *μ*_2_.

### 2.4. Dynamics of Immune Suppressors/Checkpoints

The dynamics of the immune checkpoints is given by

(4) $$\begin{array}{c} \frac{dP}{dt}=\delta -\mu_{3}P. \end{array}$$

The first term gives the source of immune checkpoints at a constant rate *δ*, and the second term is the degradation of the immune checkpoints at the rate *μ*_3_.

Finally, the interactions of the cancer cells, effector cells, BCG, and immune checkpoints together lead to the following nonlinear ordinary differential equations:

(5) dCdt=rC−α1ECP+k,dEdt=a1CEP+k+a2BEP+k−α2EC−μ1E,dBdt=b−α3EB−μ2B,dPdt=δ−μ3P,

with initial conditions *C*(0) = *C*_0_ ≥ 0, *E*(0) = *E*_0_ ≥ 0, *B*(0) = *B*_0_ ≥ 0, and *P*(0) = *P*_0_ ≥ 0.

### 2.5. Invariance of Positive Orthant

We show that the system is positively invariant.

From the system, assume *C*(0) > 0, *E*(0) > 0, *B*(0) > 0, and *P*(0) > 0.

From *dC*/*dt* = *rC* − *α*_1_*EC*/(*P* + *k*), the solution is given by *C*(*t*) = *C*_0_exp⁡(∫_0_^*t*^(*r* − *α*_1_*E*/(*P* + *k*))*dt*). This implies *C*(*t*) > 0 given that *C*_0_ > 0. Also, from *dB*/*dt* = *b* − *α*_3_*EB* − *μ*_2_*B*, if *B* = 0, then *dB*/*dt* = *b* > 0.  Therefore, *B*(*t*) > 0  ∀*t since B*_0_ > 0. Moreover, if *b* = 0, then *B*(*t*) = *B*_0_exp⁡(−∫_0_^*t*^(*α*_3_*E* + *μ*_2_)*dt*)* implying B*(*t*) > 0  ∀*t* since *B*_0_ > 0. Using *dP*/*dt* = *δ* − *μ*_3_*P*, if *δ* = 0,* then P*(*t*) = *P*_0_exp⁡(−∫_0_^*t*^*μ*_3_*dt*) > 0 since *P*_0_ > 0.

Also, if *P* = 0 and *δ* > 0,* then dP*/*dt* = *δ which implies P*(*t*) > 0  ∀*t given that P*_0_ > 0. Now consider *dE*/*dt* = (*a*_1_*C* + *a*_2_*B*)*E*/(*P* + *k*) − *α*_2_*EC* − *μ*_1_*E*, *E*(*t*) = *E*_0_ exp⁡(∫_0_^*t*^((*a*_1_*C* + *a*_2_*B*)/(*P* + *k*) − *α*_2_*C* − *μ*_1_)*dt*) > 0 given that *E*_0_ > 0.

This implies that *E*(*t*) > 0  ∀*t if E*_0_ > 0. Hence, the positive orthant *R*_+_^4^ is invariant and *C*(*t*) > 0, *E*(*t*) > 0, *B*(*t*) > 0, and *P*(*t*) > 0  ∀*t*.

## 3. Equilibrium and Stability Analysis

### 3.1. Model without Treatment (*b* = 0)

We first analyze our model in the absence of treatment (*b* = 0):

(6) dCdt=rC−α1ECP+k,dEdt=a1CEP+k+a2BEP+k−α2EC−μ1E,dBdt=−α3EB−μ2B,dPdt=δ−μ3P.

The equilibrium points of the model are obtained by equating the equations in (6) to zero and solving simultaneously for *C*, *E*, *B*, and *P*. They are as follows:

(7) U0=0,0,0,δμ3,U1=0,−μ2α3,μ1δ+kμ3a2μ3,δμ3,U2=μ1δ+kμ3a1μ3−α2δ+kμ3,rδ+kμ3α1α3,0,δμ3.

From the invariance of the positive orthant, we concentrate only on the nonnegative equilibria assuming all initial conditions are positive.

As a result, the equilibrium point *U*_1_ will not be considered. Moreover, *U*_2_ exists only if the following condition is satisfied:

(8) $$\begin{array}{c} a_{1}\mu_{3}>\alpha_{2}\left(\;\delta +k\mu_{3}\;\right). \end{array}$$

The Jacobian matrix obtained from (6) is given by

(9) $$\begin{array}{c} \hat{J}\left(\;C^{*},E^{*},B^{*},P^{*}\;\right)=\left[\begin{array}{cccc} r-\frac{\alpha_{1}E^{*}}{P^{*}+k} & \frac{-\alpha_{1}C^{*}}{P^{*}+k} & 0 & \frac{\alpha_{1}E^{*}C^{*}}{{\left(P^{*}+k\right)}^{2}}\\ \frac{a_{1}E^{*}}{P^{*}+k}-\alpha_{2}E^{*} & \frac{a_{1}C^{*}+a_{2}B^{*}}{P^{*}+k}-\alpha_{2}C^{*}-\mu_{1} & \frac{a_{2}E^{*}}{P^{*}+k} & \frac{-\left(a_{1}C^{*}E^{*}+a_{2}B^{*}E^{*}\right)}{{\left(P^{*}+k\right)}^{2}}\\ 0 & -\alpha_{3}B^{*} & -\alpha_{3}E^{*}-\mu_{2} & 0\\ 0 & 0 & 0 & -\mu_{3} \end{array}\right]. \end{array}$$

### 3.2. Stability Analysis of Equilibria of Model (6)

#### 3.2.1. Immune Checkpoints Equilibrium: *U*_0_ = {0,0, 0, *δ*/*μ*_3_}

The Jacobian matrix $\hat{J}$ evaluated at *U*_0_ yields

(10) $$\begin{array}{c} \hat{J}\left(\;U_{0}\;\right)=\left[\begin{array}{cccc} r & 0 & 0 & 0\\ 0 & -\mu_{1} & 0 & 0\\ 0 & 0 & -\mu_{2} & 0\\ 0 & 0 & 0 & -\mu_{3} \end{array}\right]. \end{array}$$

The eigenvalues of $\hat{J}\left(U_{0}\right)$ are

(11) λ1=r,λ2=−μ1,λ3=−μ2,λ4=−μ3.

Since one of the eigenvalues is always positive, then *U*_0_ is an unstable saddle point. Clinically, *U*_0_ is referred to as the death equilibrium.

#### 3.2.2. BCG-Free Equilibrium: *U*_2_ = {*μ*_1_*r*(*δ* + *kμ*_3_)^2^/(*α*_1_*a*_1_*μ*_3_^2^ − *rα*_2_(*δ* + *kμ*_3_)^2^), *r*(*δ* + *kμ*_3_)/*α*_1_*α*_3_, 0, *δ*/*μ*_3_}

Assume *U*_2_ exists; that is, *a*_1_*μ*_3_ > *α*_2_(*δ* + *kμ*_3_); then, substituting *U*_2_ in $\hat{J}$ yields the following eigenvalues:

(12) λ1=−μ3,λ2=−rα3δ+α3rkμ3+μ2α1μ3α1μ3,λ3=−δrμ3μ1+rkμ1μ32δ+kμ3,λ4=−δrμ3μ1+rkμ1μ32δ+kμ3.

Two of the eigenvalues have a real part equal to zero, which signifies neutral stability. Therefore, the equilibrium point *U*_2_ is neutrally stable.

Conclusively, in the absence of treatment, none of the equilibrium points was found to be stable.

### 3.3. Model without Immune Checkpoints

Now, we analyze the model without any suppression on the immune system by the immune checkpoints. The model is given by

(13) dCdt=rC−α1EC,dEdt=a1CE+a2BE−α2EC−μ1E,dBdt=b−α3EB−μ2B.

The equilibrium points are as follows:

(14) U0=0,0,bμ2,U1=0,ba2−μ1μ2μ1α3,μ1a2,U2=μ1μ2α1+α3rμ1−a2bα1α3ra1−α3rα2+α1μ2a1−α1α2μ2,rα1,bα1α3r+α1μ2.

The equilibrium point *U*_1_ exists only if *ba*_2_ ≥ *μ*_1_*μ*_2_. This means that the cancer cells will disappear if the constant rate of introduction of BCG and activation rate of BCG are bigger than the degradation rates of both the effector cells and the BCG.

The equilibrium point *U*_2_ also exists if

- *μ*_2_*α*_1_*μ*_1_ + *α*_3_*rμ*_1_ ≥ *a*_2_*bα*_1_  *and*  *α*_3_*a*_1_*r* + *a*_1_*α*_1_*μ*_2_ ≥ *α*_3_*ra*_2_ + *μ*_2_*α*_1_*α*_2_,
- *μ*_2_*α*_1_*μ*_1_ + *α*_3_*rμ*_1_ ≤ *a*_2_*bα*_1_  *and*  *α*_3_*a*_1_*r* + *a*_1_*α*_1_*μ*_2_ ≤ *α*_3_*ra*_2_ + *μ*_2_*α*_1_*α*_2_.

From model (13), we have the following Jacobian matrix:

(15) J−C∗,E∗,B∗=r−α1E∗−α1C∗0a1E∗−α2E∗a2B∗+a1C∗−α2C∗−μ1a2E∗0−α3B∗−α3E∗−μ2.

### 3.4. Stability Analysis of Equilibria of Model (13)

#### 3.4.1. BCG Equilibrium: *U*_0_ = {0,0, *b*/*μ*_2_}

The eigenvalues of $\overset{-}{J}$ evaluated at *U*_0_ are

(16) λ1=r,λ2=ba2−μ1μ2μ2,λ3=−μ2.

The eigenvalue *λ*_1_ is always positive and the rest are negative. Therefore, the equilibrium point *U*_0_ is an unstable saddle point.

#### 3.4.2. Cancer-Free Equilibrium: *U*_1_ = {0, (*ba*_2_ − *μ*_1_*μ*_2_)/*μ*_1_*α*_3_, *μ*_1_/*a*_2_}

Assume the equilibrium point *U*_1_ exists; then, substituting *U*_1_ in $\overset{-}{J}$ will give the following matrix:

(17) J−U1=rα3μ1−a1ba2+μ2μ1α3μ100a1ba2−a1μ2μ1−α2ba2+α2μ2μ1α3μ10a22b−a2μ2μ1α3μ10−α3μ1a2−ba2μ1.

The eigenvalues of $\overset{-}{J}\left(U_{2}\right)$ are

(18) λ1=rα3μ1+μ2μ1−a1ba2α3μ1,λ2=−ba2+ba22−4ba2μ12+4μ2μ132μ1,λ3=−ba2−ba22−4ba2μ12+4μ2μ132μ1.

Now, if

- *λ*_2_ and *λ*_3_ are complex roots, then *U*_1_ is a stable fixed point if *a*_1_*ba*_2_ > *μ*_1_(*rα*_3_ + *μ*_2_);
- *λ*_2_ and *λ*_3_ are real roots, then *U*_1_ is a stable fixed point if *ba*_2_ > *μ*_1_*μ*_2_ and *a*_1_*ba*_2_ > *μ*_1_(*rα*_3_ + *μ*_2_).

But since we already assume that the equilibrium point *U*_1_ exists, then *ba*_2_ > *μ*_1_*μ*_2_, and we can conclude that *U*_1_ is a stable fixed point if *a*_1_*ba*_2_ > *μ*_1_(*rα*_3_ + *μ*_2_).

This means that the effector cells activated by BCG will eradicate/destroy the cancer cells, if the constant rate of introduction of BCG, recruitment rate of effector cells, and the activation rate of effector cells by BCG are* bigger than or can overcome* the cancer growth rate, the rate of elimination of BCG by effector cells, and the degradation rates of effector cells and BCG altogether. Therefore, to eliminate the cancer, we* increase* the rate of introduction of BCG, rate of recruitment of effector cells, and activation rate of effector cells by BCG and at the same time* decrease* the rate of elimination of BCG by effector cells, degradation rates of both effector cells and BCG, and the cancer growth rate.

### 3.5. Model with Treatment and Immune Checkpoints

We now consider the dynamics of cancer cells, effector cells BCG, and immune checkpoints (see (5)).

The equilibrium points of model (5) are as follows:

(19) U0=0,0,bμ2,δμ3,U2=α3rδ2μ1+2α3rδμ1μ3k+α3rk2μ32μ1+μ2α1μ32μ1k+μ2α1μ3μ1δ−μ32a2bα1α3μ3rδa1−rδ2α3α2−2α3rδα2kμ3+α3rkμ32a1−α3rk2μ32α2+μ2μ32α1a1−μ2α1μ3α2δ−μ2α1μ32α2k,rδ+kμ3μ3α1,bα1μ3α3rδ+kμ3+α1μ3μ2,δμ3.

∗ The equilibrium point  U1  exists if  bμ3a2μ2μ1δ+kμ3≥1.

Also, *U*_2_ exists if

- *α*_3_*rδ*^2^*μ*_1_ + 2*α*_3_*rδμ*_1_*μ*_3_*k* + *α*_3_*rk*^2^*μ*_3_^2^*μ*_1_ + *μ*_2_*α*_1_*μ*_3_^2^*μ*_1_*k* + *μ*_2_*α*_1_*μ*_3_*μ*_1_*δ* ≥ *μ*_3_^2^*a*_2_*bα*_1_* and α*_3_*μ*_3_*rδa*_1_ + *α*_3_*rkμ*_3_^2^*a*_1_ + *μ*_2_*μ*_3_^2^*α*_1_*a*_1_ ≥ *rδ*^2^*α*_3_*α*_2_ + 2*α*_3_*rδα*_2_*kμ*_3_ + *α*_3_*rk*^2^*μ*_3_^2^*α*_2_ + *μ*_2_*α*_1_*μ*_3_*α*_2_*δ* + *μ*_2_*α*_1_*μ*_3_^2^*α*_2_*k*;
- *α*_3_*rδ*^2^*μ*_1_ + 2*α*_3_*rδμ*_1_*μ*_3_*k* + *α*_3_*rk*^2^*μ*_3_^2^*μ*_1_ + *μ*_2_*α*_1_*μ*_3_^2^*μ*_1_*k* + *μ*_2_*α*_1_*μ*_3_*μ*_1_*δ* ≤ *μ*_3_^2^*a*_2_*bα*_1_* and α*_3_*μ*_3_*rδa*_1_ + *α*_3_*rkμ*_3_^2^*a*_1_ + *μ*_2_*μ*_3_^2^*α*_1_*a*_1_ ≤ *rδ*^2^*α*_3_*α*_2_ + 2*α*_3_*rδα*_2_*kμ*_3_ + *α*_3_*rk*^2^*μ*_3_^2^*α*_2_ + *μ*_2_*α*_1_*μ*_3_*α*_2_*δ* + *μ*_2_*α*_1_*μ*_3_^2^*α*_2_*k*.

From model (5), we obtain the following Jacobian matrix:

(20) $$\begin{array}{c} \tilde{J}\left(\;C^{*},E^{*},B^{*},P^{*}\;\right)=\left[\begin{array}{cccc} r-\frac{\alpha_{1}E^{*}}{P^{*}+k} & \frac{\alpha_{1}C^{*}}{P^{*}+k} & 0 & \frac{\alpha_{1}E^{*}C^{*}}{{\left(P^{*}+k\right)}^{2}}\\ \frac{a_{1}E^{*}}{P^{*}+k}-\alpha_{2}E^{*} & \frac{a_{1}C^{*}+a_{2}B^{*}}{P^{*}+k}-\alpha_{2}C^{*}-\mu_{1} & \frac{a_{2}E^{*}}{P^{*}+k} & -\frac{\left(a_{1}E^{*}C^{*}+a_{1}B^{*}C^{*}\right)}{{\left(P^{*}+k\right)}^{2}}\\ 0 & -\alpha_{3}B^{*} & -\alpha_{3}E^{*}-\mu_{2} & 0\\ 0 & 0 & 0 & -\mu_{3} \end{array}\right]. \end{array}$$

### 3.6. Stability Analysis of Equilibria of Model (5)

#### 3.6.1. BCG and Immune Checkpoints Equilibrium: *U*_0_ = {0,0, *b*/*μ*_2_, *δ*/*μ*_3_}

The eigenvalues of $\tilde{J}$ evaluated at *U*_0_ are

(21) λ1=r,λ2=bμ3a2−μ2μ1δ−μ2μ1μ3kμ2δ+kμ3,λ3=−μ2,λ4=−μ3.

Since one of the eigenvalues is always positive, then *U*_0_ is an unstable saddle point.

#### 3.6.2. Tumor-Free Equilibrium: *U*_1_ = {0, (*bμ*_3_*a*_2_ − *μ*_2_*μ*_1_*δ* − *μ*_2_*μ*_1_*kμ*_3_)/*μ*_1_*α*_3_(*δ* + *kμ*_3_), *μ*_1_(*δ* + *kμ*_3_)/*μ*_3_*a*_2_, *δ*/*μ*_3_}

Assume this equilibrium point exists; then, the eigenvalues of $\tilde{J}$ evaluated at *U*_1_ are as follows:

(22) λ1=−μ3,λ2=α3rδ2μ1+2α3rδμ3μ1k+α3rk2μ32μ1+μ2α1μ32μ1k+μ2α1μ3μ1δ−μ32a2bα1α3μ1δ+kμ32,λ3=−bμ3a2+bμ3a22+4μ13δ2μ2+8μ13δμ2kμ3+4μ32μ13k2μ2−4μ12δbμ3a2−4μ32μ12kba22μ1δ+μ3k,λ4=−bμ3a2−bμ3a22+4μ13δ2μ2+8μ13δμ2kμ3+4μ32μ13k2μ2−4μ12δbμ3a2−4μ32μ12kba22μ1δ+μ3k.

The equilibrium point *U*_1_ is a stable fixed point if

(23) a2bμ3μ1μ2δ+kμ3>max⁡1,rμ3kα3+μ3μ2α1+α3rδμ2α1μ3.

However, condition ∗ is already true; then, *U*_1_ is a stable fixed point if

(24) $$\begin{array}{c} \frac{a_{2}b\mu_{3}}{\mu_{1}\mu_{2}\left(\delta +k\mu_{3}\right)}>\frac{\left(r\mu_{3}k\alpha_{3}+\mu_{3}\mu_{2}\alpha_{1}+\alpha_{3}r\delta \right)\mu_{2}}{\alpha_{1}\mu_{3}}. \end{array}$$

#### 3.6.3. Interior Equilibrium: *U*_2_ = {*C*^*∗*^, *r*(*δ* + *kμ*_3_)/*μ*_3_*α*_1_, *bα*_1_*μ*_3_/(*α*_3_*r*(*δ* + *kμ*_3_) + *α*_1_*μ*_3_*μ*_2_), *δ*/*μ*_3_}

The eigenvalues of the Jacobian matrix $\tilde{J}(U_{2})$ are very long, complicated, and difficult to analyze. Therefore, we use numerical simulations to show the stability of the equilibrium point *U*_2_.

## 4. Numerical Illustrations

In this section, the numerical simulations of the three models will be shown. The aim here is to show the effect of immune checkpoints on the effector cells. We use MATLAB version 2016b to plot the graphs with initial populations of the compartments involved taken to be equal. Other parameters used in the numerical simulations are given in Table 1.

**Figure 1:**
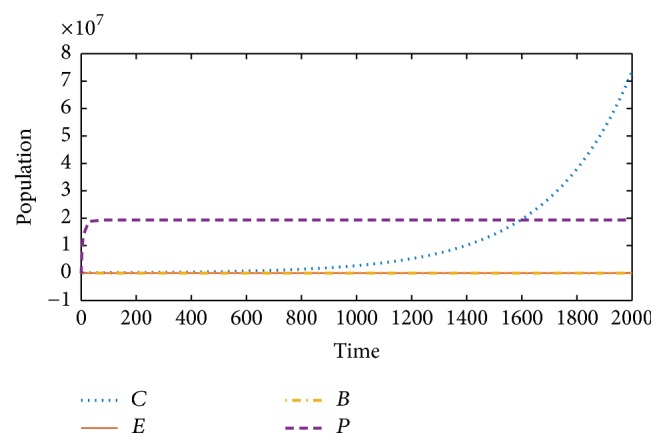
Model (6) (without treatment): cancer cells (*C*) grow exponentially, overcoming the effector cells (*E*), with the help of immune checkpoints (*P*).

We first plot the graph of model (6) to illustrate what happens in the absence of treatment. As expected, the cancer cells develop with the help of suppression on the effector cells by the immune checkpoints, hence dominating the effector cells and resulting in the growth and maturation of the cancer. Therefore, the numerical simulations of model (6) support this notion as shown in Figure 1.

**Figure 2:**
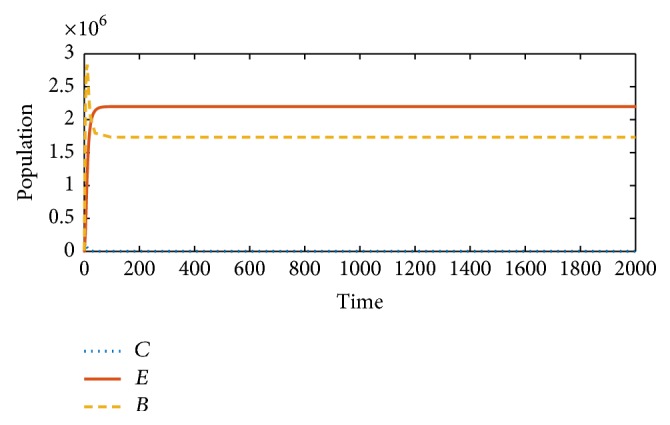
Model (13) (without immune suppressors): the effector cells (*E*) overcome the development of cancer cells (*C*) as a result of the stimulation and activation by the BCG (*B*).

Next, we show the behavior of model (13) (i.e., without the immune checkpoints). Here, we will see how the effector cells attack and kill the cancer cells as a result of the stimulation/activation by the BCG. Unlike in Figure 1, Figure 2 shows how the growth of the cancer cells is restricted and eventually leads to their extinction by the effector cells.

**Figure 3:**
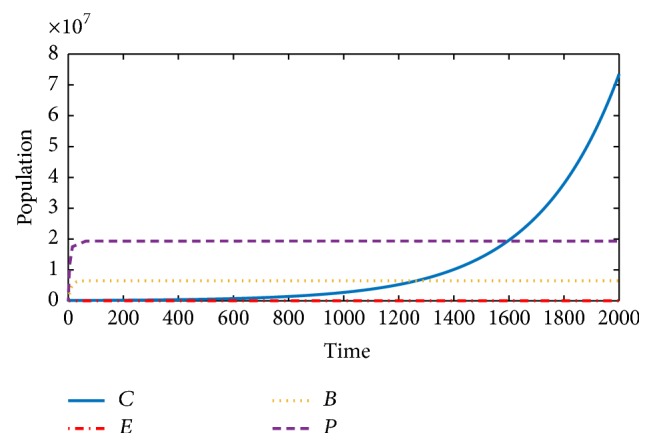
Model (5): despite the stimulation of effector cells (*E*) by the BCG (*B*), the immune checkpoints (*P*) block and deactivate the activities of the effector cells, thereby leading to the development and progression of cancer cells (*C*).

The general model will now be considered. Despite stimulation and activation of the effector cells by the BCG, the immune suppressors block and deactivate their function; hence, this leads to the reduction of autoimmunity of the effector cells. Therefore, the cancer develops and grows exponentially as shown in Figure 3.

Therefore, comparing Figures 2 and 3, we will notice the effect of immune checkpoints on the effector cells. In Figure 2, the effector cells in the absence of immune suppressors fight the cancer cells, resulting in stopping their development and progression, while Figure 3 shows the progression and development of the cancer cells as a result of the presence of immune suppressors.

## 5. Conclusion and Discussion

In this paper, we used a system of four nonlinear ordinary differential equations to model the dynamics of cancer cells, effector cells, BCG, and immune checkpoints/suppressors in the immunotherapy of bladder cancer. We derived three possible dynamics from our model. Firstly, the model was analyzed in the absence of treatment and we studied the stability analysis of the equilibria involved. Figure 1 shows how the cancer progressed in the absence of treatment and presence of immune checkpoints/suppressors.

Secondly, we study the model without the immune checkpoints/suppressors. Conditions for stability of the equilibria involved were also given. In the absence of immune checkpoints/suppressors, the activated-effector cells have unlimited freedom to roam about and detect the cancer cells; as a result, they kill them and stop the cancer from progressing. This was shown in Figure 2.

Thirdly, we considered the dynamics of the model with treatment and the immune checkpoints/suppressors. Conditions for stability of the equilibrium points were given, and Figure 3 shows how the cancer cells grow and develop despite the application of the treatment (BCG). This is believed to be as a result of the blockage and suppression that the effector cells suffered by the immune checkpoints.

Therefore, the figures used in this paper assist in showing the effect of immune checkpoints/suppressors on the effector cells and the treatment at large. To avoid cancer progression and advancement, there is a need for action to block or limit the production of the immune checkpoints. This will take the brakes off the immune system and thereby allow it to mount a stronger and more effective attack against cancer cells.

Nivolumab is a drug recently approved by the FDA to be used alone or with other drugs to treat cancer. It is a fully human immunoglobulin (Ig) G4 monoclonal antibody directed against the negative immunoregulatory human cell surface receptor programmed cell death protein-1 (PD-1) with immune checkpoint inhibitory and antineoplastic activities. Nivolumab binds to and blocks the activation or production of immune checkpoints like PD-1. This results in the activation of T-cells and cell-mediated responses against cancer cells. So, the primary role of nivolumab is to block the immune checkpoints from suppressing the immune systems. Hence, this helps in allowing the immune cells to rise against cancer cells without any interference [16, 17].

## Figures and Tables

**Table 1:** List of all parameters used in numerical simulations.

Parameter	Interpretation (units)	Estimated value	Reference
*r*	Tumor growth rate *t*^−1^ = day^−1^	0.0033	Shochat et al., 1999
*α*_1_	Rate of elimination of cancer cells by effector cells cell day^−1^	1.1 × 10^−7^	Kuznetsov et al., 1994
*k*	Inhibitory parameter	2 × 10^3^	Not found
*a*_1_	Recruitment rate of effector cells *t*^−1^ = day^−1^	0.25	Sud D. et al., 2006
*a*_2_	Activation rate of effector cells by the BCG cells^−1^ day^−1^	0.052	Wigginton and Kirschner, 2001
*δ*	Internal production of immune checkpoints	1.51932 × 10^5^	Sandip Banerjee et al., 2015
*α*_2_	Elimination rate of effector cells by cancer cells cells^−1^ day^−1^	3.45 × 10^−10^	Kuznetsov et al., 1994
*μ*_1_	Degradation rate of effector cells *t*^−1^ = day^−1^	0.041	Kuznetsov et al., 1994
*μ*_2_	Rate of BCG decay *t*^−1^ = day^−1^	0.1	Archuleta et al., 2002
*b*	Bioeffective concentration of BCG c.f.u./day	6.5 × 10^5^	Cheng et al., 2004
*α*_3_	Destruction of BCG by effector cells cells^−1^ day^−1^	1.25 × 10^−7^	Wigginton and Kirschner, 2001
*μ*_3_	Degradation rate of immune checkpoints *t*^−1^ = day^−1^	166.32	Sandip Banerjee et al., 2015
